# Mechanical Stimulation Modulates Osteocyte Regulation of Cancer Cell Phenotype

**DOI:** 10.3390/cancers13122906

**Published:** 2021-06-10

**Authors:** Stefaan W. Verbruggen, Clare L. Thompson, Michael P. Duffy, Sophia Lunetto, Joanne Nolan, Oliver M. T. Pearce, Christopher R. Jacobs, Martin M. Knight

**Affiliations:** 1 Department of Biomedical Engineering, Columbia University in the City of New York, New York, NY 10027, USA; michael.p.duffy@columbia.edu (M.P.D.); christopher.jacobs@columbia.edu (C.R.J.); 2 Centre for Predictive in vitro Models, School of Engineering and Materials Science, Queen Mary University of London, London E1 4NS, UK; clare.l.thompson@qmul.ac.uk (C.L.T.); s.lunetto@qmul.ac.uk (S.L.); joanne.nolan@qmul.ac.uk (J.N.); m.m.knight@qmul.ac.uk (M.M.K.); 3 Department of Mechanical Engineering and INSIGNEO Institute for in silico Medicine, University of Sheffield, Sheffield S1 3JD, UK; 4 Queen Mary + Emulate Organs-on-Chips Centre, Queen Mary University of London, London E1 4NS, UK; 5 Barts Cancer Institute, School of Medicine and Dentistry, Queen Mary University of London, London EC1M 5PZ, UK; o.pearce@qmul.ac.uk

**Keywords:** breast cancer, prostate cancer, tumour metastasis, bone metastatic disease, cell co-culture, mechanical stimulation, osteocyte, organ on a chip, microfluidic device

## Abstract

**Simple Summary:**

Metastatic lesions in bone tissue are a common complication in breast and prostate cancer patients, accounting for the larger part of pain and suffering in late-stage cancer. The metastatic cancer cells that form these lesions must travel from the primary tumour to a distant bone and enter a mechanically active environment that is largely regulated in response to physical exercise by bone cells known as osteocytes. This study used cell culture techniques to investigate if osteocytes can regulate breast and prostate cancer cells, and how mechanical stimulation of these sensitive bone cells affects cancer cell behaviour. Osteocytes signalled for decreased proliferation of cancer cells, but mechanical stimulation reversed this in breast cancer. By developing a microfluidic organ-chip model, we demonstrated the feasibility and importance of replicating the mechanical tumour microenvironment, finding increased invasion of cancer cells with mechanical stimulation.

**Abstract:**

Breast and prostate cancers preferentially metastasise to bone tissue, with metastatic lesions forming in the skeletons of most patients. On arriving in bone tissue, disseminated tumour cells enter a mechanical microenvironment that is substantially different to that of the primary tumour and is largely regulated by bone cells. Osteocytes, the most ubiquitous bone cell type, orchestrate healthy bone remodelling in response to physical exercise. However, the effects of mechanical loading of osteocytes on cancer cell behaviour is still poorly understood. The aim of this study was to characterise the effects of osteocyte mechanical stimulation on the behaviour of breast and prostate cancer cells. To replicate an osteocyte-controlled environment, this study treated breast (MDA-MB-231 and MCF-7) and prostate (PC-3 and LNCaP) cancer cell lines with conditioned media from MLO-Y4 osteocyte-like cells exposed to mechanical stimulation in the form of fluid shear stress. We found that osteocyte paracrine signalling acted to inhibit metastatic breast and prostate tumour growth, characterised by reduced proliferation and invasion and increased migration. In breast cancer cells, these effects were largely reversed by mechanical stimulation of osteocytes. In contrast, conditioned media from mechanically stimulated osteocytes had no effect on prostate cancer cells. To further investigate these interactions, we developed a microfluidic organ-chip model using the Emulate platform. This new organ-chip model enabled analysis of cancer cell migration, proliferation and invasion in the presence of mechanical stimulation of osteocytes by fluid shear stress, resulting in increased invasion of breast and prostate cancer cells. These findings demonstrate the importance of osteocytes and mechanical loading in regulating cancer cell behaviour and the need to incorporate these factors into predictive in vitro models of bone metastasis.

## 1. Introduction

Breast and prostate are the two most prevalent cancer types worldwide, with the vast majority of these 1 million combined annual deaths related to metastatic disease [1]. Bone tissue is generally one of the most common tumour metastasis sites, with over 450,000 patients currently suffering from this condition in the US [2]. This is particularly true for breast and prostate cancer, with 65–75% of breast and prostate metastatic patients developing skeletal lesions, together accounting for more than 80% of all cases of metastatic bone disease [3,4]. Furthermore, we now know that metastatic spread is an early event in breast cancer development [5,6], with disseminated tumour cells already detectable in bone marrow by the time primary tumours are found [7]. Once tumour metastasis has been diagnosed, five-year patient survival rates in the UK decrease from 90–98% when diagnosed at Stage I-II to 26% at Stage IV for breast cancer, and 99% to 30% at equivalent stages for prostate cancer [8]. Upon discovery of bone metastases, prognosis is particularly poor, with a median survival time of 1–4 years in breast and prostate cancer patients [3]. Therefore, bone metastases are now the largest contributor to patient suffering and mortality for breast and prostate cancer patients, and yet how these tumours establish a presence in bone remains poorly understood.

Clinical trials have suggested that combined aerobic and resistance exercise programs result in reliable and meaningful improvements in quality of life, fatigue, aerobic fitness, bone mineral density and muscular strength in breast cancer patients [9,10]. Mechanical loading (i.e., load-bearing physical exercise) has also been found to decrease metastasis-induced osteolysis in a xenograft model [11]. Similarly, a systematic meta-analysis of exercise in prostate cancer patients found improvements in quality of life, cancer-specific fatigue, strength and fitness [12], though no changes to metastasis were observed and bone metastases actually increased in one exercise group [13]. In contrast to breast cancer research, no study has applied mechanical loading to an in vivo model of prostate cancer metastases in bone tissue, possibly due to the lack of in vitro studies into this mechanical microenvironment.

To date, much of the literature has focused on interactions between disseminated tumour cells, extracellular matrix components in the tumour microenvironment [14,15], and the varied bone cell types residing in the bone marrow [16,17] and vascular niches [18]. Indeed, a number of recent studies suggest that osteoblast-like cells may arise within primary breast [19] and prostate tumours [20]. While this approach is inherently sensible, given the marrow is the likely point of arrival of tumour cells, it neglects more than 90% of bone cells [21]. These cells, osteocytes, reside within the bone matrix in a series of interconnected cavities and channels, known as the lacunocanalicular network [22,23]. Within this network, osteocytes act as master regulators of bone health through a variety of mechanisms. These include coordinating the activity of osteoblasts, osteoclasts and other cell types to balance bone deposition and resorption [21,24,25]; sensing and responding to mechanical forces [26,27,28,29,30]; actively remodelling the surrounding bone matrix [31,32,33]. Importantly, osteocytes have been observed to upregulate cytokine signalling in vitro in response to applied mechanical loading, in the form of both substrate stretch and fluid flow [34,35,36]. Since cytokines are potent regulators of cancer cell behaviour, it is therefore possible that mechanical stimulation of osteocytes associated with physical exercise, may alter cancer cell metastasis.

Despite the important role of osteocytes in healthy bone remodelling, their potential interactions with cancer cells are relatively understudied. A number of previous studies have identified the role of the osteocyte in regulating cancer cell behaviour, finding that osteocyte paracrine signalling can alter proliferative, migratory, and invasive behaviours [37,38,39,40,41]. Notably, no study has yet examined the effect of osteocyte mechanical stimulation on prostate cancer cells, with breast cancer remaining the primary focus of study. A key limitation in this area has been the difficulty in selectively loading osteocytes while in co-culture with cancer cells, which has confined researchers to conditioned media studies. This has been partially overcome with the advent of microfluidic co-culture organ-chip platforms, with a number of models recently developed in attempts to recapitulate the bone marrow and bone tissue microenvironments [42,43,44]. This technology has been applied to replicate extravasation of MDA-MB-231 breast cancer cells in the presence of osteocytes, neatly demonstrating reduced extravasation of these cells with mechanical stimulation of the bone cells [37]. Therefore, this developing technology presents a novel platform to investigate the effects of mechanical stimulation and bone–cancer interactions in vitro.

Osteocytes have been unveiled as a potential key player in the metastatic cascade in both breast and prostate cancer, with mechanical stimulation an important factor in this developing tumour microenvironment. Therefore, the aim of this study was to investigate the influence of mechanically stimulated osteocytes on the proliferation, invasion and migration potential of a selection of common breast and prostate cancer cell lines. Additionally, a co-culture organ-chip system is presented, providing a more predictive in vitro model of osteocyte–cancer cell cross-talk and regulation by mechanical loading during the formation of metastatic tumours.

## 2. Materials and Methods

### 2.1. Cell Culture Conditions

The MLO-Y4 osteocyte-like mouse cell line, a kind gift from Professor L. Bonewald (University of Missouri, Kansas City, MO, USA), was cultured on collagen-coated surfaces (rat tail collagen type I, 0.15 mg/mL) with α-modified essential medium (α-MEM, Thermofisher, Waltham, MA, USA) supplemented with 2.5% foetal bovine serum (FBS), 2.5% iron supplemented calf serum (CS, HyClone Laboratories, Logan, UT, USA), and 100 U/mL penicillin and 100 μg/mL streptomycin (all Sigma-Aldrich, St. Louis, MO, USA). Two human breast cancer cell lines (MDA-MB-231 and MCF-7), and two human prostate cancer cell lines (PC3 and LNCaP) were sourced from the American Type Culture Collection (ATCC), and were routinely maintained in Dulbecco’s modified Eagle’s medium (DMEM, Thermofisher) supplemented with 10% FBS, and 100 U/mL penicillin and 100 μg/mL streptomycin (all Sigma-Aldrich). All cells were maintained at 37 °C, with 5% CO^2^ and 95% humidity. Conditioned media (CM) was collected after 48 h of culture on osteocytes, then centrifuged for 10 min at 10,000 rpm and vacuum-filtered through Steriflip 0.22 µm filters (Sigma-Aldrich) to remove suspended cells and cellular debris.

Mechanical loading was applied to the MLO-Y4 cells using oscillatory fluid flow generated by culturing cells in rectangular flasks (82 × 92 mm; 10 mL of media) on a rocking platform which oscillated at a frequency of 0.5 Hz and with an amplitude of 1.5 cm for 24 h after an initial 24 h static period post seeding. This system has been shown to generate spatiotemporal fluid-flow induced maximal shear stress of approximately 0.1 Pa across a layer of cells [45,46] that is partially representative of that experienced by osteocytes within the lacunar network in bone (0.01–1 Pa) [47,48,49,50]. In all experiments, CM was collected after 24 h of fluid shear or unsheared static culture conditions.

In all cases, CM from MLO-Y4 cells was applied to cancer cells at a 1:1 ratio. Uncultured MLO-Y4 standard media was applied at a 1:1 ratio to cancer cells in control groups, to remove any variability from combining different media types. Each conditioned media experiment contained 3 sample wells, and was repeated on 3 separate occasions, resulting in *n* = 9 samples per group.

### 2.2. Microfluidic Organ-Chip Culture Conditions

The design and fabrication of the Organ-Chip (S-1, Emulate Inc., Boston, MA, USA) were described previously [51]. Briefly, the chip is composed of a flexible polydimethylsiloxane (PDMS) elastomer containing two parallel microchannels (1 × 1 mm and 1 × 0.2 mm, cancer cell and osteocyte channel, respectively) [52] separated by a porous flexible PDMS membrane (50 μm thick, with 7 μm diameter pores with 40 μm spacing, giving 2% porosity over a surface area of 0.171 cm^2^ separating the two channels). Both channels of the chips were coated with collagen I (rat tail collagen type I, 0.15 mg/mL; Sigma). Coated chips were then incubated overnight at 37 °C and 5% CO^2^. Chips were inverted and seeded in the bottom channel with MLO-Y4 osteocyte-like cells at 1,500,000 cells/mL, which were allowed to attach to the membrane over 2 h before flushing with fresh media. Chips were then flipped upright, and the procedure repeated to seed the top channel with cancer cells at a concentration of 400,000 cells/mL. Cells were allowed to acclimatise to culture conditions for 24 h.

Mechanical stimulation to the MLO-Y4 cells was provided by increasing the media flow rate in the osteocyte channel. The standard flow rate for media replenishment was set at 30 μL/h, applying a negligible shear stress of ~3 × 10^−5^ Pa, while the mechanically loaded channels were assigned a flow rate of 1000 μL/h, generating a shear stress across the osteocytes of approximately 0.03 Pa. This high flow condition was applied every day for 6 h, for 10 days post-seeding.

### 2.3. Proliferation Assay

Cancer cell proliferation was assessed using the AlamarBlue cell viability assay (Life Technologies, Eugene, OR, USA) which detected redox reduction during cell growth. In each experiment, cancer cells were seeded onto 24-well plates at a density of 25 × 10^3^ cells/cm^2^. At the experimental endpoint, after 48 h cultured with control or conditioned media, 50 μL of the AlamarBlue reagent was added to each well containing cells and 500 μL of culture medium. Cells were then incubated for 3 h at 37 °C. The fluorescence was measured with a Synergy 4 multi-mode microplate reader (BioTek Instruments, Winooski, VT, USA) with excitation at 544 nm and emission at 590 nm. The fluorescence value was proportional to the number of viable cells, and absolute values for these data are contained in supplementary information (Figure S2).

In the microfluidic chips, individual cells were counted for each field of view obtained in order to quantify the degree of cell proliferation in the presence or absence of mechanical stimulation.

### 2.4. Invasion Assays

Invasiveness of cancer cells was measured using an in vitro Matrigel invasion assay [53]. Briefly, transwell inserts (8-μm pores) for 24-well plates were precoated with 50 μL/insert of 1 mg/mL Matrigel (Corning Inc., Corning, NY, USA), for 1 h at 37 °C. Subsequently, cancer cells were seeded into the upper chamber of each insert at 75 × 10^3^ cells/cm^2^ in 250 μL basal medium. Then, 500 μL of either control medium or CM was added to the lower chamber under the inserts. After incubation for 24 h, cells that had penetrated the Matrigel-coated membrane and adhered to other side of the inserts were dissociated with Trypsin (Sigma-Aldrich) for 7 min at 37 °C. A total of 250 mL of media was then added to neutralise the Trypsin. AlamarBlue was then added to the solution containing invaded cells, with the assay performed as described for proliferation above. Absolute values for invasion experiments are contained in supplementary information (Figure S3).

Within the chip model, invasion of cancer cells through two matrix layers and across the porous chip membrane was measured by staining with EpCAM, which is strongly expressed by breast and prostate cancer cells, but not expressed by MLO-Y4 cells. EpCAM stain in the bone channel was quantified using ImageJ.

### 2.5. Migration Assays

Migration assays were performed over a 12 h period on both the conditioned media and chip co-culture experiments, which is substantially lower than the >24 h doubling times of each cell line. A 24-well plate was seeded with cancer cells at a density of 50 × 103 cells/cm^2^ and cultured until formation of a confluent monolayer. After 48 h of CM treatment, the monolayer was scratched with a 200 µL pipette tip to create a linear wound approximately 200 μm wide. Migration of the cells into the wound gap was monitored by light microscopy serial time-lapse imaging for 12 h using a Lumascope LS720 live-cell imaging system (Etaluma Inc., Carlsbad, CA, USA) with a 10× objective. Cells remained in conditioned media throughout this period. The percentage of wound gap closure was measured using ImageJ software (National Institutes of Health, Bethesda, MD, USA) as previously described [54].

The chip model is a closed microenvironment preventing use of the scratch wound assay. Therefore, cell migration was measured using a standard cell migration tracking plugin for ImageJ. Cells were stained for 45 min with CellTracker Green (Catalogue # C7025, Thermofisher Scientific, Waltham, MA, USA) at 5 µM in serum-free medium, and were then trypsinised and seeded as normal, with the stain remaining visible for up to 9 days. Migration across the surface of the membrane of 20 individual cells in each chip was measured on time-lapse images captured every hour over a 12 h period on day 8, with time-lapse scans obtained using the Muvicyte Live-Cell Imaging System (PerkinElmer, Waltham, MA, USA). Absolute values for gap closure are included in supplementary data (Figure S4).

### 2.6. Immunocytochemistry and Microscopy

Cells in both channels on chips were stained for DAPI (1 ug/mL) and Alexa Fluor-647 conjugated Phalloidin (both ThermoFisher Scientific), as well as cancer cell-specific marker EpCAM (CD326, Catalogue #53-8326-42, Thermofisher Scientific). Imaging was performed at 20× on a Zeiss 710 ELYRA PS.1 confocal microscope using an EC Plan-Neofluar10×/0.3 M27 objective (Zeiss, Oberkochen, Germany). Confocal z-sections were made throughout the cell depth (approximately 20 sections) using 5 μm step size with an image format of 2048 × 2048 yielding a pixel size of 0.415 × 0.415 μm (image size approximately 850 × 850 μm).

### 2.7. Statistical Analysis

As described in figure legends, the statistical analyses were performed using one-way ANOVA with Bonferroni post-hoc test using GraphPad Prism 5 (GraphPad Software, San Diego, CA, USA). Statistical significance compared to associated controls indicated as follows: * *p* < 0.05, ** *p* < 0.05, *** *p* < 0.001, by one-way ANOVA with Bonferroni post-hoc test. Where indicated in the figure legends, conditioned media experiments were repeated independently multiple times and similar results were obtained.

## 3. Results

### 3.1. Proliferation in All Cancer Cell Lines Was Decreased by Osteocyte Conditioned Media, with Mechanical Stimulation Reversing This Effect in Breast Cancer Cells

**Figure 1:**
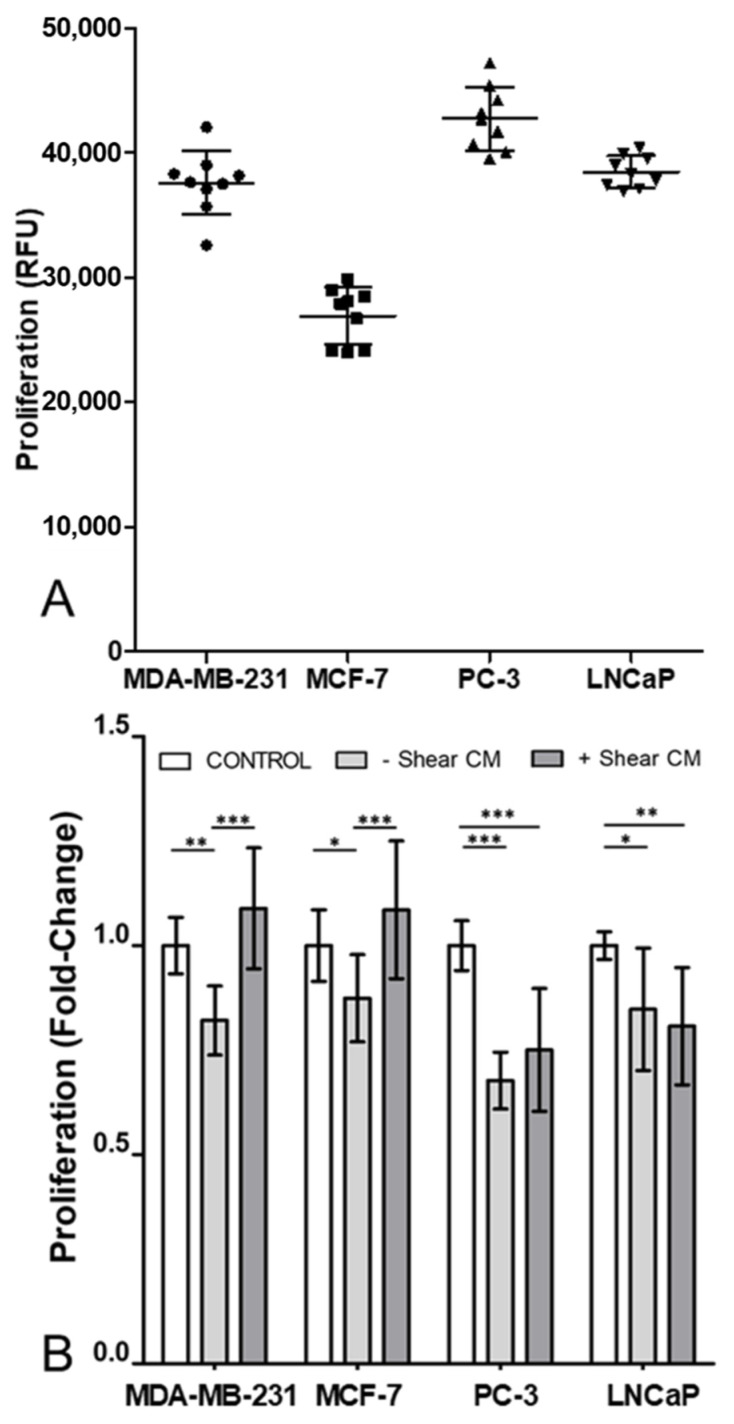
Osteocyte conditioned media decreased proliferation in all cell lines. Mechanical stimulation of osteocytes reversed this decrease in breast cancer cells, but not prostate cancer cells. (**A**) Absolute values of proliferation, in relative fluorescence units, of breast (MDA-MD-231 and MCF-7) and prostate (PC-3 and LNCaP) cancer cell lines, after 48 h in standard control media. (**B**) Fold change in proliferation relative to control showing the effect of 50% diluted conditioned media from MLO-Y4 osteocyte-like cells under no-shear or flow-shear conditions (*n* = 9). Data normalised to control after 48 h with bar representing mean ± standard deviation. Statistically significant differences are indicated based on one-way ANOVA with Bonferroni post-hoc test (* *p* < 0.05, ** *p* < 0.01, *** *p* < 0.001).

The triple-negative breast cancer cell line MDA-MB-231 was observed to have higher proliferation than the oestrogen receptor-positive MCF-7 breast cancer cell line, while among the two prostate cancer cell lines the androgen receptor-negative PC-3 cells proliferated more rapidly than the receptor-positive LNCaP cells (Figure 1A). Addition of unstimulated conditioned media from MLO-Y4 osteocyte-like cells resulted in significantly reduced proliferation, in both breast cancer cell lines and both prostate cancer cell lines (Figure 1B). Media taken from MLO-Y4 cells mechanically stimulated via fluid shear, reversed this effect in breast cancer cells. This was shown by a significant increase in proliferation compared to breast cancer cells treated with conditioned media from unloaded osteocytes. Consequently, there were no significant differences in breast cancer cell proliferation compared to cells without conditioned media. However, in prostate cancer cells, fluid shear-loading of osteocytes did not change proliferation when compared to cells treated with conditioned media from unloaded osteocytes (Figure 1B).

### 3.2. Invasion of Cancer Cells Was Decreased by Osteocyte Conditioned Media with and without Mechanical Stimulation

**Figure 2:**
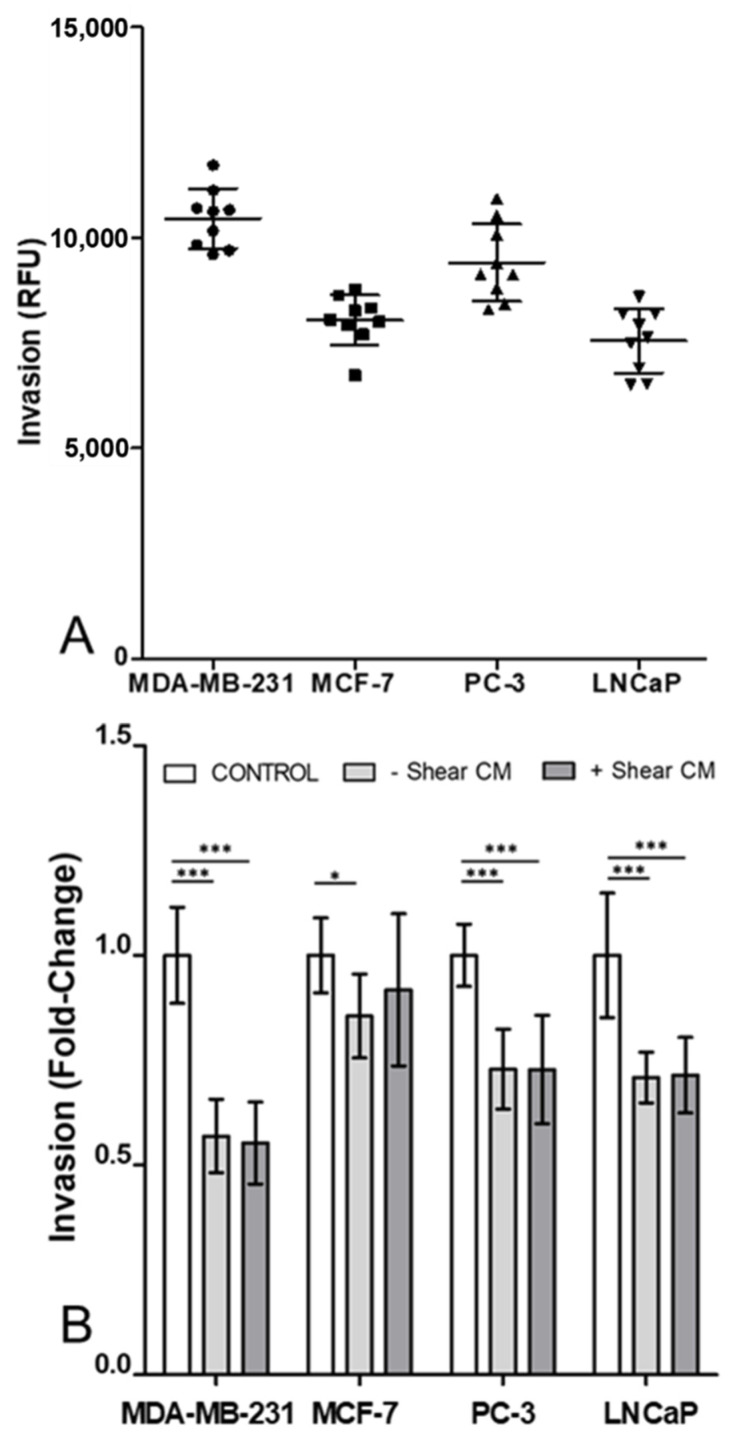
Osteocyte conditioned media decreased invasion in both breast and prostate cell lines, regardless of application of mechanical stimulation. (**A**) Absolute values of invasion through Matrigel-coated transwell inserts, in relative fluorescence units, of breast (MDA-MD-231 and MCF-7) and prostate (PC-3 and LNCaP) cancer cell lines, after 48 h in standard control media. (**B**) Fold change in invasion relative to control showing the effect of 50% diluted conditioned media from MLO-Y4 osteocyte-like cells under unloaded or fluid-shear conditions (*n* = 9). Data normalised to control after 48 h with bar representing mean ± standard deviation. Statistically significant differences are indicated based on one-way ANOVA with Bonferroni post-hoc test (* *p* < 0.05, *** *p* < 0.001).

Absolute values for invasion were broadly similar for most of the cell lines, apart from the more invasive MDA-MB-231 cells (Figure 2A). In a similar manner to proliferation, osteocyte conditioned media broadly inhibited invasion in all four cancer cell types (Figure 2B). Mechanical stimulation of osteocytes via fluid shear had no effect on this response in any of the cancer cell lines, such that there were no statistically significant differences in invasion levels with and without shear.

### 3.3. Migration in Three of the Four Cancer Cell Lines Was Increased by Osteocyte Conditioned Media, with Mechanical Stimulation of Osteocytes Reversing this Effect

**Figure 3:**
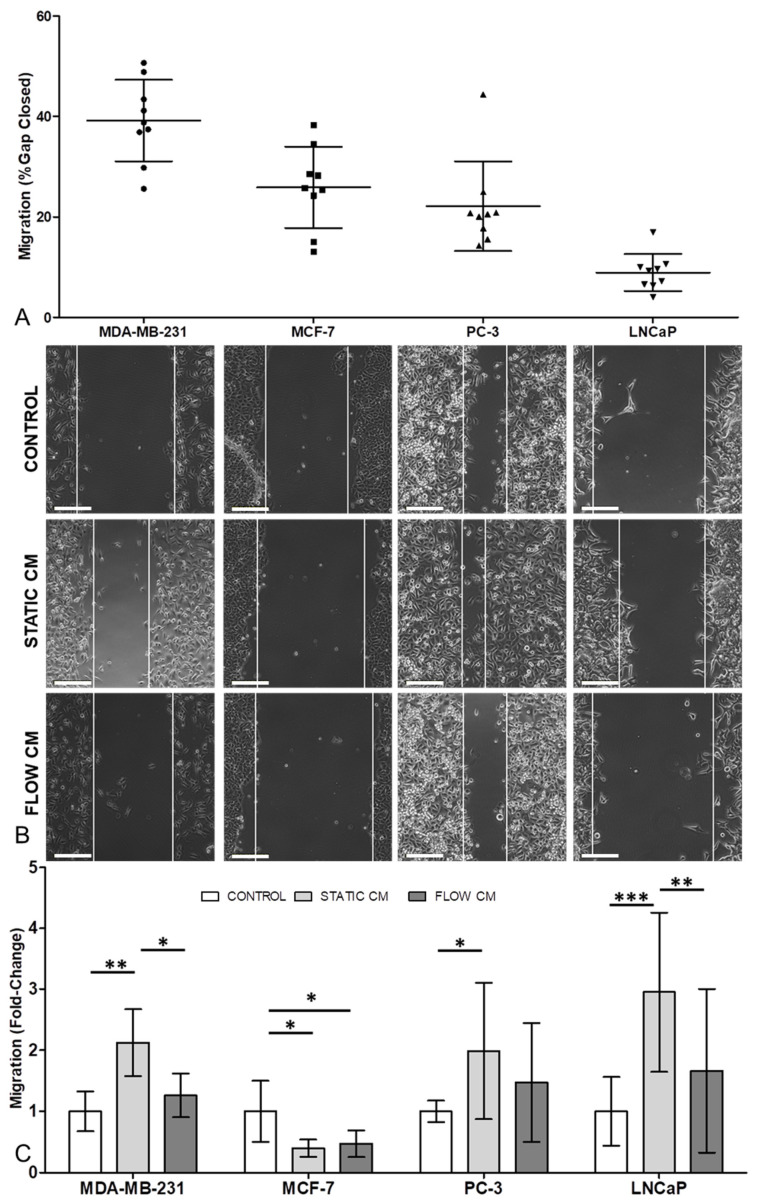
Osteocyte conditioned media increased migration in one of the breast and both of the prostate cancer cells lines, with mechanical stimulation of osteocytes reversing this effect. (**A**) Absolute values of migration of breast (MDA-MD-231 and MCF-7) and prostate (PC-3 and LNCaP) cancer cell lines, after 48 h in standard control media. (**B**) Representative images of observed migration 12 h post scratch-wound assay in MDA-MB-231, MCF-7, PC-3 and LNCaP cancer cell lines. Scale bar = 50 µm. (**C**) Fold change in migration relative to control showing the effect of 50% diluted conditioned media from MLO-Y4 osteocyte-like cells under static or fluid-shear conditions (*n* = 9). Data normalised to control after 48 h with bar representing mean ± standard deviation. Statistically significant differences are indicated based on one-way ANOVA with Bonferroni post-hoc test (* *p* < 0.05, ** *p* < 0.01, *** *p* < 0.001).

The most migratory cells were the MDA-MB-231 breast cancer line and the least migratory were the LNCaP prostate cancer line, with MCF-7 and PC-3 cells migrating by similar amounts (Figure 3A). Conditioned media treatment of cancer cells resulted in large increases in migration in one breast (MDA-MB-231) and both prostate cancer cell lines (Figure 3B,C). In these cell lines, mechanically stimulated osteocyte conditioned media partly reversed this switch to a more migratory phenotype. By contrast, the application of osteocyte condition media to the MCF-7 breast cancer cell line reduced migration behaviour both with and without osteocyte mechanical stimulation.

### 3.4. An Organ-Chip Co-culture Model Replicated Effect of Conditioned Media on Cancer Cell Migration but Not Proliferation or Invasion

**Figure 4:**
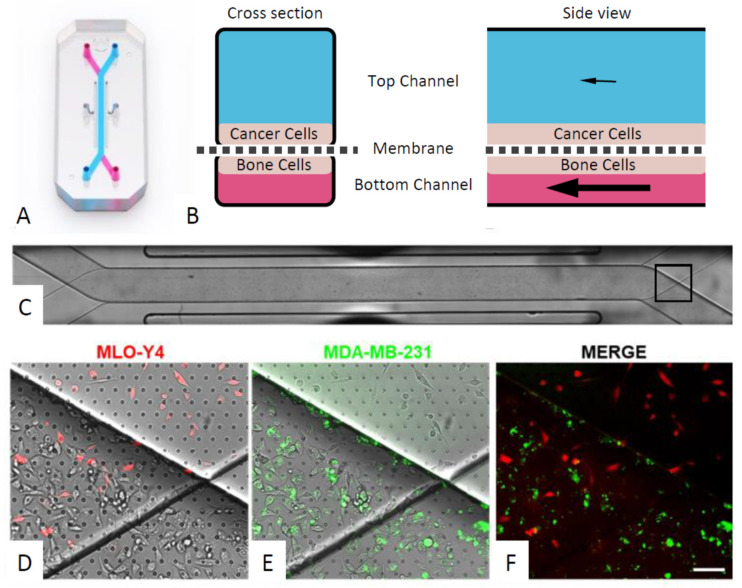
The microfluidic organ-chip incorporating bone and cancer cell channels. (**A**) The Emulate organ-chip (reproduced with permission). (**B**) Schematics showing the cancer cells in the top channel (1 × 1 mm) and the bone cells in the bottom channel (1 × 0.2 mm) subjected to fluid shear stress of 0.03 Pa caused by media flow. The two channels are separated by a membrane with 7 µm diameter pores. (**C**) Brightfield image showing the entire length of the channels. The boxed region at the right-hand end is magnified to show MLO-Y4 cells in the bottom channel (**D**, red) and MDA-MB-231 cells in the top channel (**E**, green) and merged (**F**). Scale bar = 20 µm.

We successfully developed an organ-chip co-culture model using the Emulate platform. This enabled MLO-Y4 cells to be stimulated with fluid shear in one channel and separated by a porous membrane from either breast (MDA-MB-231) or prostate (PC-3) cancer cells in the other channel (Figure 4).

**Figure 5:**
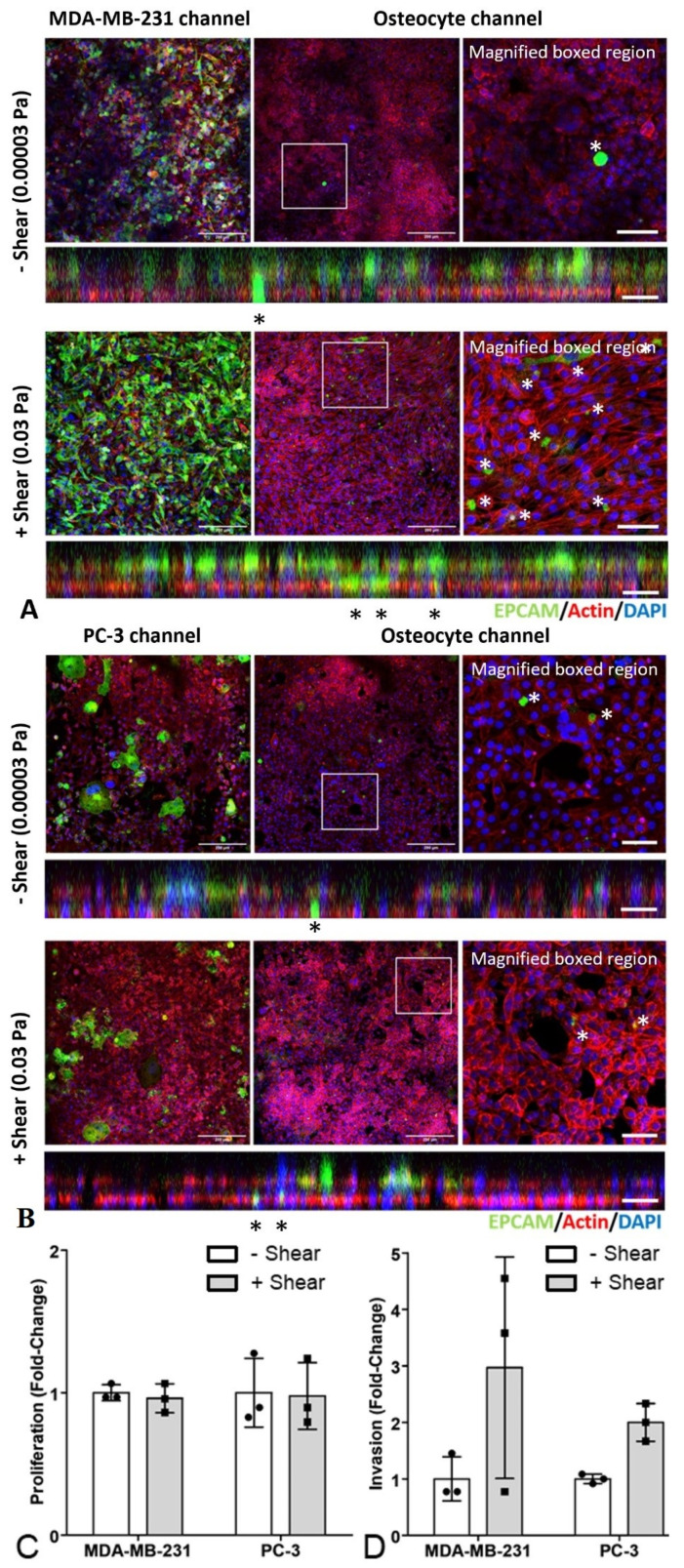
Mechanical stimulation of osteocytes had no effect on proliferation but increased invasion in organ-chip models of breast and prostate cancer bone metastasis. Representative confocal images of (**A**) the MDA-MB-231 breast cancer bone metastasis chip and (**B**) the PC-3 prostate cancer bone metastasis chip. Immunofluorescent staining for cancer cells (EpCAM, green), actin (Phalloidin, red) and nuclei (DAPI, blue) in both the cancer cell channel and the osteocyte channel. The images are single confocal planes with an additional magnified view of the osteocyte channel (right) and the orthogonal projection showing both channels (below). The upper panels show images from organ chips in which the MLO-Y4 cells were exposed to minimal shear stress (0.00003 Pa), with lower panels showing organ-chips with higher shear conditions (0.03 Pa). Invasion of EpCAM-stained cancer cells through the porous membrane into the bone channel are indicated (*). Scale bar = 20 µm. Associated analysis of EpCAM-positive cancer cells to quantify (**C**) proliferation in both channels, and (**D**) invasion into the bone channel. Data based on three separate confocal z-stacks for each chip.

Using this organ-chip model, data from a single chip experimental found that mechanical loading of MLO-Y4 cells via fluid shear (0.03 Pa) had minimal effect on proliferation of either MDA-MB-231 or PC-3 cells. Fluid shear had no effect on proliferation of either cancer cell type as quantified by number of EpCAM stained cells in the top channel (Figure 5) but appeared to trigger increased invasion of breast and prostate cancer cells (EpCAM stained cells in bottom channel).

**Figure 6:**
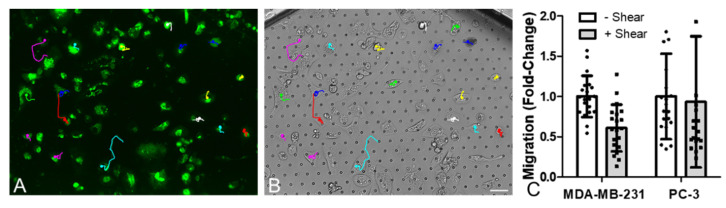
Mechanical stimulation of osteocytes in organ-chip models decreased migration of breast cancer cells but had no effect on prostate cancer cells. (**A**) Representative confocal image showing the technique for quantification of migration of cancer cells as measured via live-cell imaging and tracking of cells labelled with CellTracker (green). (**B**) The corresponding bright-field image (scale bar = 20 µm). (**C**) Associated quantification of migration for individual cells within chips (*n* = 20–40 cells from 1 organ-chip per group).

Fluid shear inhibited MDA-MB-231 cell migration, but not PC-3 cell migration, replicating effects seen with conditioned media (Figure 6). These preliminary results in the organ-chip model differ from those seen with conditioned media in terms of cancer cell proliferation and invasion.

## 4. Discussion

The use of cell lines within in vitro models presents a number of limitations. The different origins of the mouse MLO-Y4 osteocyte-like cells, and the human breast and prostate cancer cells, is not ideal. However, these are very well-established cancer cell lines, which are frequently studied in mouse models of cancer cell metastasis [55,56]. The MLO-Y4 cell line is limited in its low sclerostin expression, a Wnt signalling inhibitor, relative to osteocytes in vivo. Despite this, MLO-Y4 cells are by far the most well-understood osteocyte cell line [57] and have the advantage of a more consistent phenotype compared to other options, such as primary cell isolations. Use of MLO-Y4 cells also allows for comparison to the few other studies in this nascent area of research. Therefore, while acknowledging the limitations of these cell lines, their use allowed for tight control of experimental variabilities and direct comparison with similar studies. 

Throughout this study mechanical stimulation of osteocytes was applied via specific quantifiable levels of fluid shear stress to replicate the in vivo loading environment within bone [47,48,49,50]. However, it is important to note the difference in the loading regimes employed in the conditioned media experiments (Figure 1, Figure 2 and Figure 3) versus the co-culture developed in the organ-chip model (Figure 4, Figure 5 and Figure 6). For conditioned media experiments, we used oscillatory shear stress of 0.1 Pa whilst in the organ-chip the mechanical loading was provided as unidirectional shear stress of 0.03 Pa. The loading in the organ-chip is the maximum shear stress currently achievable using the Emulate platform which can normally only apply unidirectional flow in each channel. Furthermore, while the shear on osteocytes due to the rocking experiment did not disrupt the stable osteocyte monolayer or visually affect osteocyte cell number, the conditioned media has not been normalised to subtle changes in cell number in these experiments. Additionally, in a preliminary experiment on a single chip, osteocyte number was not found to change with application of shear due to flow (Figure S1).

This study examines the effects of osteocyte paracrine signalling on two breast cancer cell lines (MDA-MB-231 and MCF-7) and two prostate cancer cell lines (PC-3 and LNCaP). Soluble factors released from the bone cells downregulate proliferation (Figure 1) and invasion (Figure 2) whilst, in all but the MCF-7 cells, conditioned media from the osteocytes increased migration (Figure 3). Together these findings indicate that osteocytes encourage metastatic cancer cells towards a more mesenchymal and less proliferative phenotype.

A number of previous studies have similarly identified the role of the osteocyte in regulating cancer cell behaviour, finding that osteocyte paracrine signalling can alter proliferative, migratory, and invasive behaviours [37,38,39,40,41]. This has been most widely studied using breast cancer cell lines. A study by Cui et al. was the first to investigate this experimentally, using conditioned media to demonstrate that soluble factors secreted by osteocytes could upregulate proliferation and migration in a range of breast and prostate cancer cell lines [39]. This work was expanded upon to include the effect of mechanical loading, showing that the triple-negative MDA-MB-231 breast cancer cell line exhibited reduced invasiveness and trans-endothelial migration when treated with flow-stimulated osteocyte conditioned media [38,41]. A separate study into the oestrogen receptor-positive MCF-7 breast cancer cell line identified a new potential mechanism, CXCL1/2, through which mechanical stimulation of osteocytes may upregulate proliferation and migration in these cells [40]. However, these studies have reported conflicting findings. Whilst some studies report increased proliferation and transwell invasion of MDA-MB-231 cells with conditioned media [39], and further increases in invasion with loading [38], other studies report decreased MCF-7 migration and proliferation with applied fluid shear [40]. Nevertheless, it is clear from these previous studies and our data presented here that osteocytes regulate breast cancer cell behaviour and that aspects of this regulation are modulated by mechanical loading of the osteocytes. However, this behaviour appears to be sensitive to differences in experimental set-up, making direct comparisons with previous studies challenging.

Intriguingly, mechanical stimulation of osteocytes by fluid shear inhibited the regulatory effect of conditioned media on breast cancer cell proliferation but not invasion. This effect of loading was particularly evident in MDA-MB-231 breast cancer cells, where conditioned media from loaded osteocytes completely blocked the reduction in proliferation and increase in migration otherwise seen without loading. Interestingly, using our organ-chip co-culture model, mechanical loading of osteocytes stimulated invasion in these MDA-MB-231 breast cancer cells. 

In this study we also present new data for prostate cancer cells, demonstrating for the first time the effects of signalling from osteocytes and how this is regulated by mechanical loading. Compared to breast cancer cells, conditioned media experiments indicated that mechanical loading had minimal effect on prostate cancer cells. However, the preliminary findings from our organ-chip co-culture model showed that fluid shear stimulation of osteocytes triggers increased invasion of both breast and prostate cancer cells (Figure 5). Together our data suggests that fluid shear forces on osteocytes, as generated by mechanical loading during exercise, may block the normal osteocyte suppression of metastasis in breast and prostate cancer. 

From a clinical perspective it is interesting that applied loading led to decreased invasion in all cell lines, as this would imply a protective effect for mechanical loading via exercise of bone tissue. However, in breast cancer cells, flow stimulation decreased migration and increased proliferation, indicating that loading of osteocytes signals for more proliferative behaviour in breast cancer cells that could enhance tumour growth. The contrasting behaviour observed in prostate cancer cells under the influence of osteocytes may underlie the osteoblastic lesions commonly found in prostate cancer patients, with breast cancer metastatic patients more frequently presenting with osteolytic lesions. Indeed, while this study focused on the regulation of cancer cells by osteocyte mechanobiology, our observations of reduced proliferation of MLO-Y4 cells in a chip imply that cancer cells can regulate osteocyte behaviour in turn. This has important implications for degenerative diseases such as osteoporosis, as these conditions have previously been shown to result in decreased mechanosensitivity in osteocytes in aged and osteoporotic bone [28,48,58]. Additional disruption of osteocyte mechanosensation by cytokine signalling from cancer cells would likely exacerbate this effect, and further degrade bone quality.

This research area is likely to benefit from the expanding use of organ-chip models such as the Emulate platform. This enabled controlled levels of fluid shear stress to be applied to bone cells within a microfluidic channel whilst monitoring cancer cell behaviour in a separate channel connected by a porous membrane. In so doing, the organ-chip models replicate a more realistic tumour microenvironment in order to better understand how bone cells regulate cancer cell behaviour and metastasis. 

## 5. Conclusions

Further work is needed to investigate the possible reciprocal interaction between cancer cells and the various bone cell types, as well as the impact of physical exercise on breast and prostate metastasis in vivo. However, this study presents important evidence showing that mechanical stimulation is a potent regulator of osteocyte–cancer cell interactions in the developing metastatic cascade, for both breast and prostate cancer. We found that osteocyte signalling generally inhibits metastatic breast and prostate tumour growth, but that mechanical stimulation may reverse some of these effects. Indeed, our co-culture, organ-chip model demonstrates increased breast and prostate cancer cell invasion with mechanical stimulation of osteocytes. This study therefore highlights both the feasibility and the importance of including mechanical stimulation within predictive organ-chip and other in vitro models of metastatic cancer in bone.

## Data Availability

The data presented in this study are available in this article and Supplementary Materials.

## Supplementary Materials

The following are available online at https://www.mdpi.com/article/10.3390/cancers13122906/s1, Figure S1: Fold change in proliferation of MLO-Y4 osteocyte cells under loading in organ-chip, Figure S2: Absolute values in relative fluorescence units for proliferation of breast (MDA-MB-231, MCF-7) and prostate (PC-3, LNCaP) cancer cells when treated with control media, and conditioned media from loaded or unloaded MLO-Y4 osteocytes, Figure S3: Absolute values in relative fluorescence units for invasion of breast (MDA-MB-231, MCF-7) and prostate (PC-3, LNCaP) cancer cells when treated with control media, and conditioned media from loaded or unloaded MLO-Y4 osteocytes, Figure S4. Absolute values for percentage gap closure in a scratch wound migration assay for breast (MDA-MB-231, MCF-7) and prostate (PC-3, LNCaP) cancer cells when treated with control media, and conditioned media from loaded or unloaded MLO-Y4 osteocytes.
