# Supplementary material for: Mechanical Stimulation Modulates Osteocyte Regulation of Cancer Cell Phenotype
Source: Cancers (Basel). 2021 Jun 10;13(12):2906. doi: 10.3390/cancers13122906 (PMC8230361; doi:10.3390/cancers13122906)
Supplement: Supplementary file 1 [file cancers-13-02906-s001.zip › cancers-1202882-SI.pdf]

cancers

MDPI

Supplementary Materials: Mechanical stimulation modulates osteocyte regulation of cancer cell phenotype

Stefaan W. Verbruggen, Clare L. Thompson, Michael P. Duffy, Sophia Lunetto, Joanne Nolan, Oliver M.T. Pearce, Christopher R. Jacobs and Martin M. Knight

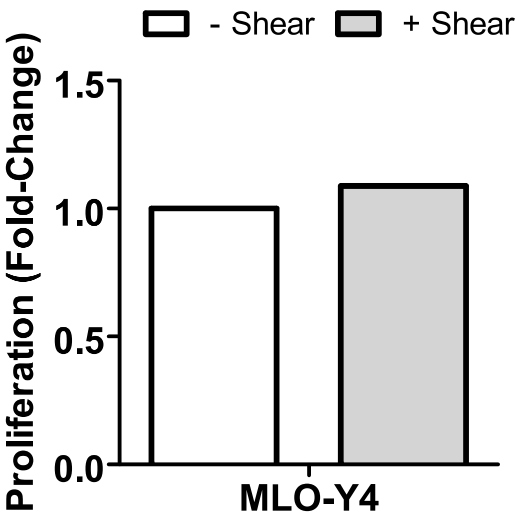

Figure S1. Fold change in proliferation of MLO-Y4 osteocyte cells under loading in organ-chip ($n = 1$ chip per group).

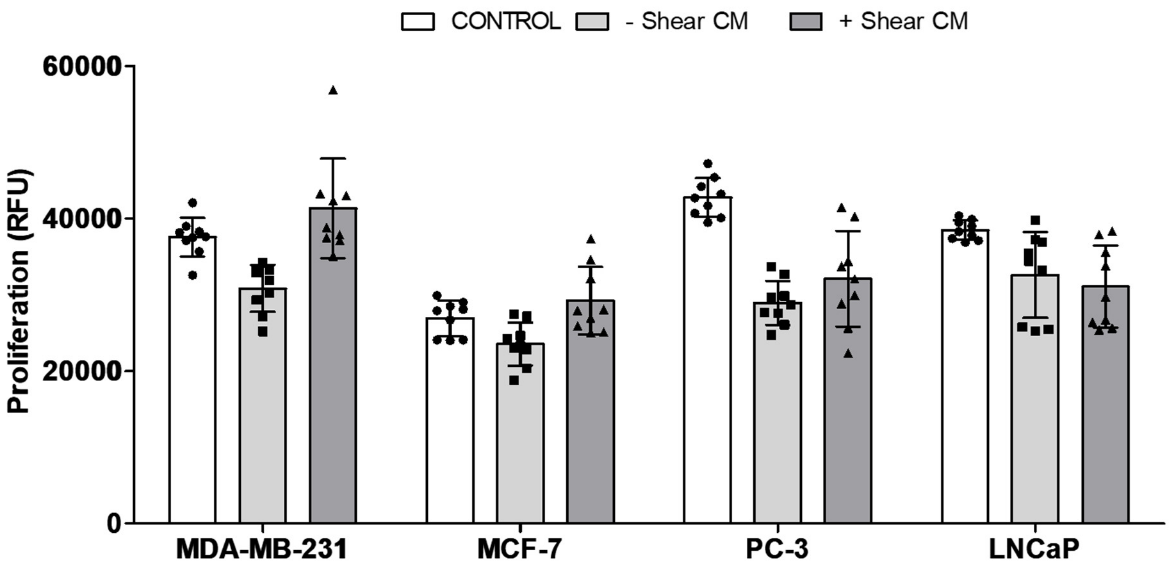

Figure S2. Absolute values in relative fluorescence units for proliferation of breast (MDA-MB-231, MCF-7) and prostate (PC-3, LNCaP) cancer cells when treated with control media, and conditioned media from loaded or unloaded MLO-Y4 osteocytes ($n = 9$, 3 experiments with 3 replicates).

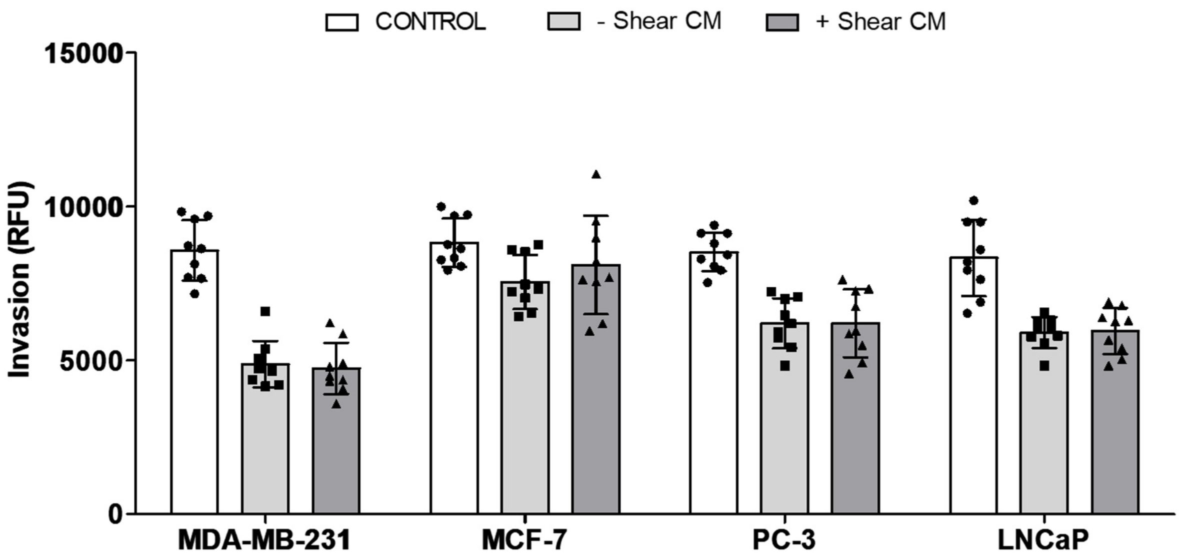

Figure S3. Absolute values in relative fluorescence units for invasion of breast (MDA-MB-231, MCF-7) and prostate (PC-3, LNCaP) cancer cells when treated with control media, and conditioned media from loaded or unloaded MLO-Y4 osteocytes (n = 9, 3 experiments with 3 replicates).

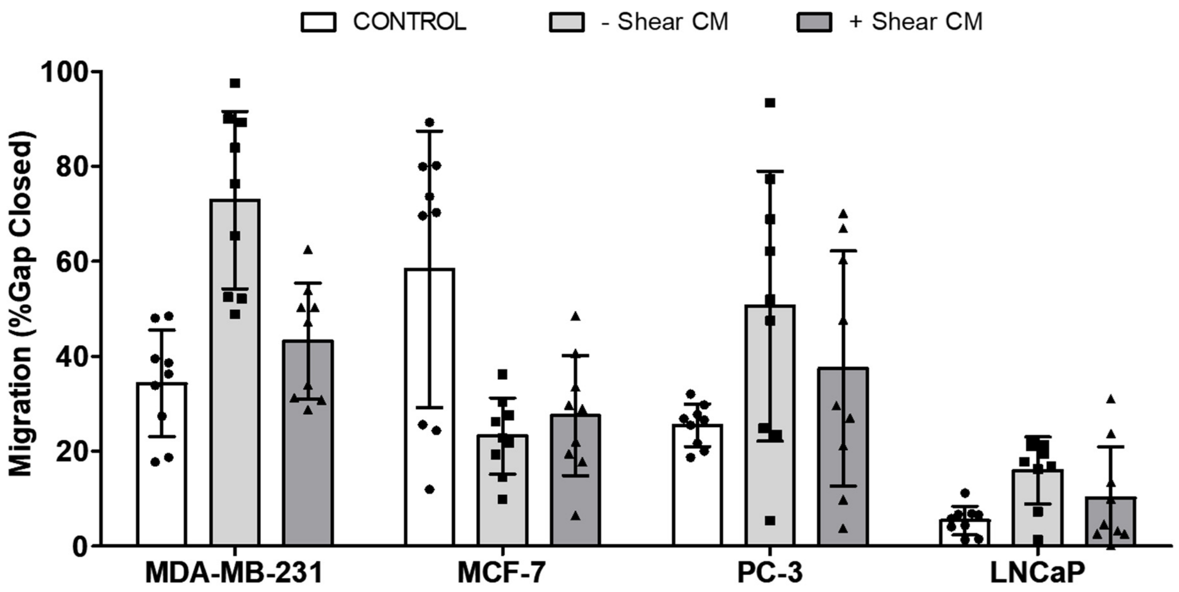

Figure S4. Absolute values for percentage gap closure in a scratch wound migration assay for breast (MDA-MB-231, MCF-7) and prostate (PC-3, LNCaP) cancer cells when treated with control media, and conditioned media from loaded or unloaded MLO-Y4 osteocytes (n = 9, 3 experiments with 3 replicates).
